# Supplementary material for: Two KaiABC systems control circadian oscillations in one cyanobacterium
Source: Nat Commun. 2024 Sep 3;15:7674. doi: 10.1038/s41467-024-51914-5 (PMC11372060; doi:10.1038/s41467-024-51914-5)
Supplement: Supplementary file 1 — Supplementary information [file 41467_2024_51914_MOESM1_ESM.pdf]

Supplementary Information for

Two KaiABC systems control circadian oscillations in one cyanobacterium

Christin Köbler[a,1], Nicolas M. Schmelling[b,1], Anika Wiegard[b,1], Alice Pawlowski[b,1], Gopal K. Pattanayak[c], Philipp Spät[d], Nina M. Scheurer[a], Kim N. Sebastian[a], Florian P. Stirba[b], Lutz C. Berwanger[b], Petra Kolkhof[b], Boris Maček[d], Michael J. Rust[c], Ilka M. Axmann[b,]*, Annegret Wilde[a,]*

[a]Institute of Biology III, Faculty of Biology, University of Freiburg, 79104 Freiburg, Germany; [b]Institute for Synthetic Microbiology, Biology Department, Heinrich Heine University Düsseldorf, 40225 Düsseldorf, Germany; [c]Department of Molecular Genetics and Cell Biology, The University of Chicago, Chicago, IL 60637, United States of America; [d]Department of Quantitative Proteomics, Interfaculty Institute for Cell Biology, Eberhard Karls University Tübingen, 72076 Tübingen, Germany

[1]C.K., N.M.S, A. Wie, and A.P. contributed equally to this study.

Corresponding authors:

*Prof. Dr. Annegret Wilde, Albert-Ludwigs-Universität Freiburg, Institut für Biologie III, Schänzlestr. 1, 79104 Freiburg, Germany, Phone: +49 (0) 761-20397828

*Prof. Dr. Ilka Axmann, Institute for Synthetic Microbiology, Biology Department, Heinrich Heine University Düsseldorf, 40225 Düsseldorf, Germany, Phone: +49 (0) 21181-10361

Email: annegret.wilde@biologie.uni-freiburg.de; Ilka.Axmann@hhu.de

This PDF file includes:

Figures S1 to S11
Table S1

This manuscript includes Supplementary Data S1 to S4

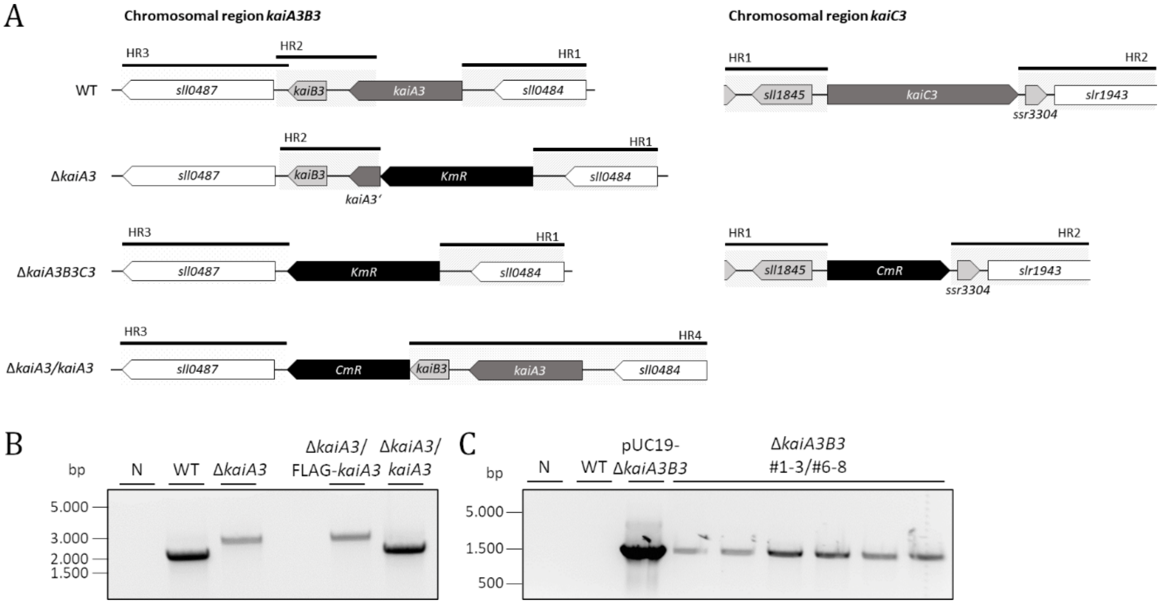

Fig. S1. Construction of mutants of the KaiC3 based clock system. (A) Schematic depiction of the *kaiA3B3 and* kaiC3 genomic context. Gene locus of *kaiA3B3* with the up- and downstream located genes. *KaiA3* and *kaiB3* are transcribed as an operon together with *sll0484*, the putative promoter is upstream of *sll0484*. To inactivate *kaiA3*, the gene was replaced with a kanamycin resistance cassette (KmR). To construct the triple knockout mutant Δ*kaiAB3C3*, the genomic region from *kaiA3* to *kaiB3* was replaced with a kanamycin resistance cassette (KmR) in the Δ*kaiC3* strain. Complementation of the Δ*kaiA3* strain was achieved by the introduction of *kaiA3* within its original genomic context with a chloramphenicol resistance cassette introduced downstream of the *kaiB3* gene. Clones were selected for chloramphenicol resistance (CmR) and lack of kanamycin resistance. Black bars and grey, dashed boxes represent the regions for homologous recombination into the *Synechocystis* chromosome. (B) Representative result for the verification of the complete segregation of the Δ*kaiA3* deletion strain and Δ*kaiA3*/*kaiA3* complementation strain using colony PCR with the oligonucleotides P15/P19 and P20/P29 (Table S1A). A non-template reaction (N), chromosomal WT DNA and Δ*kaiA3*/FLAG-*kaiA3* served as control reactions. Expected construct sizes are 1913 bp for the WT allele and Δ*kaiA3*/*kaiA3*, and 2472 bp for Δ*kaiA3* and Δ*kaiA3*/FLAG-*kaiA3*. (C) Verification of the *kaiA3B3* deletion in the Δ*kaiC3* strain using colony PCR with the oligonucleotides P15/P30. A non-template reaction (N), chromosomal WT DNA, and the vector pUC19-Δ*kaiA3B3* served as controls. Expected construct size for Δ*kaiA3B3* and pUC19-Δ*kaiA3B3* is 1554 bp. No construct was expected for the WT allele. Complete segregation was verified using oligonucleotides P19/P30 (not shown).

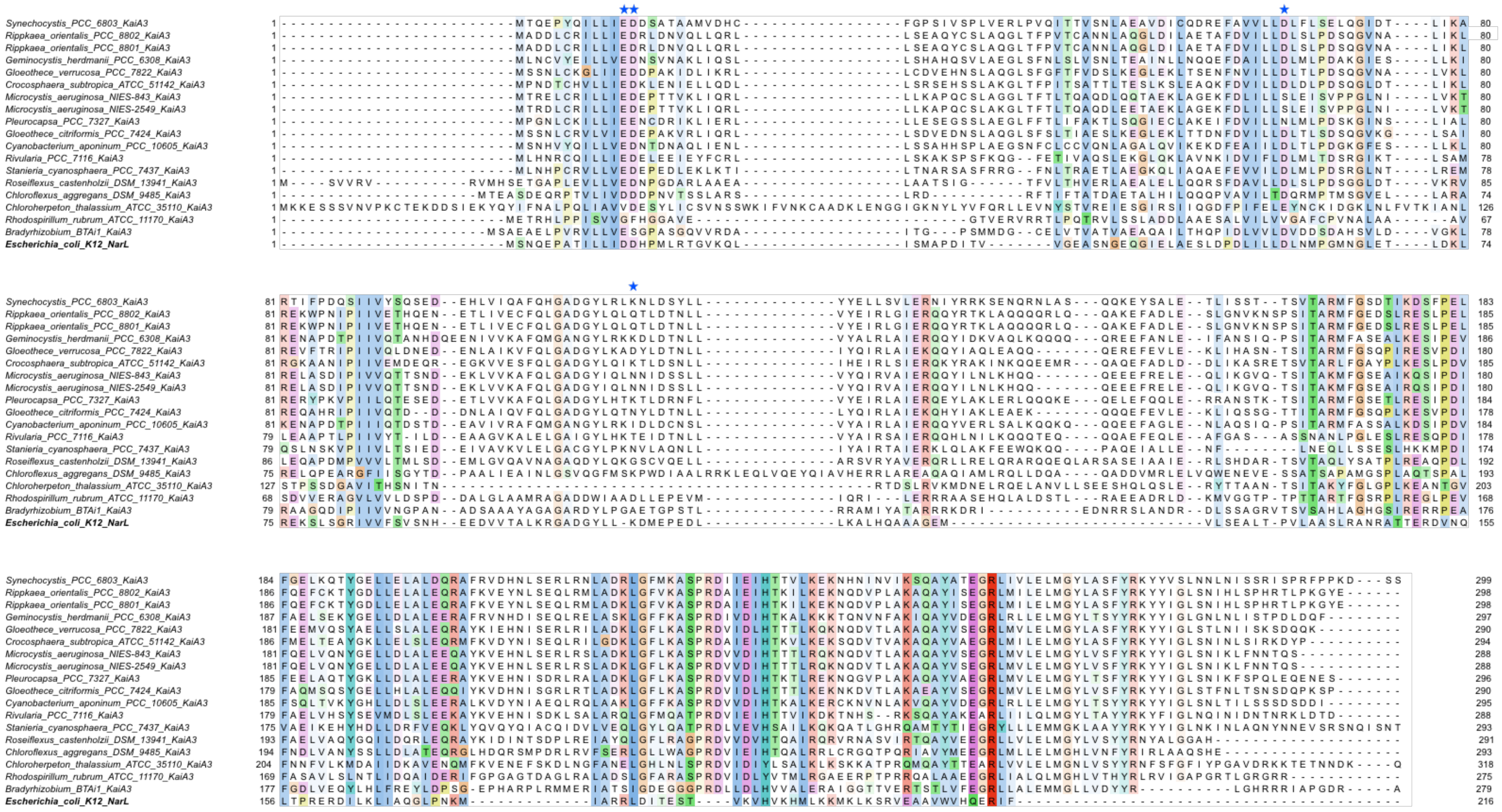

Fig. S2. Alignment of the amino acid sequences of Sll0485 (KaiA3) orthologs including NarL from *E. coli*. The sequences were aligned with Mafft (preset, L-INS-i). Sequences are represented in the Clustalx color code with conservation visibility set 20 %[1, 2, 3]. As a representative of NarL-type response regulators, the NarL homolog of the *E. coli* strain K12 (UniProtKB - P0AF28) was added. The residues crucial for phosphorylation in response regulators are marked with a blue star[4].

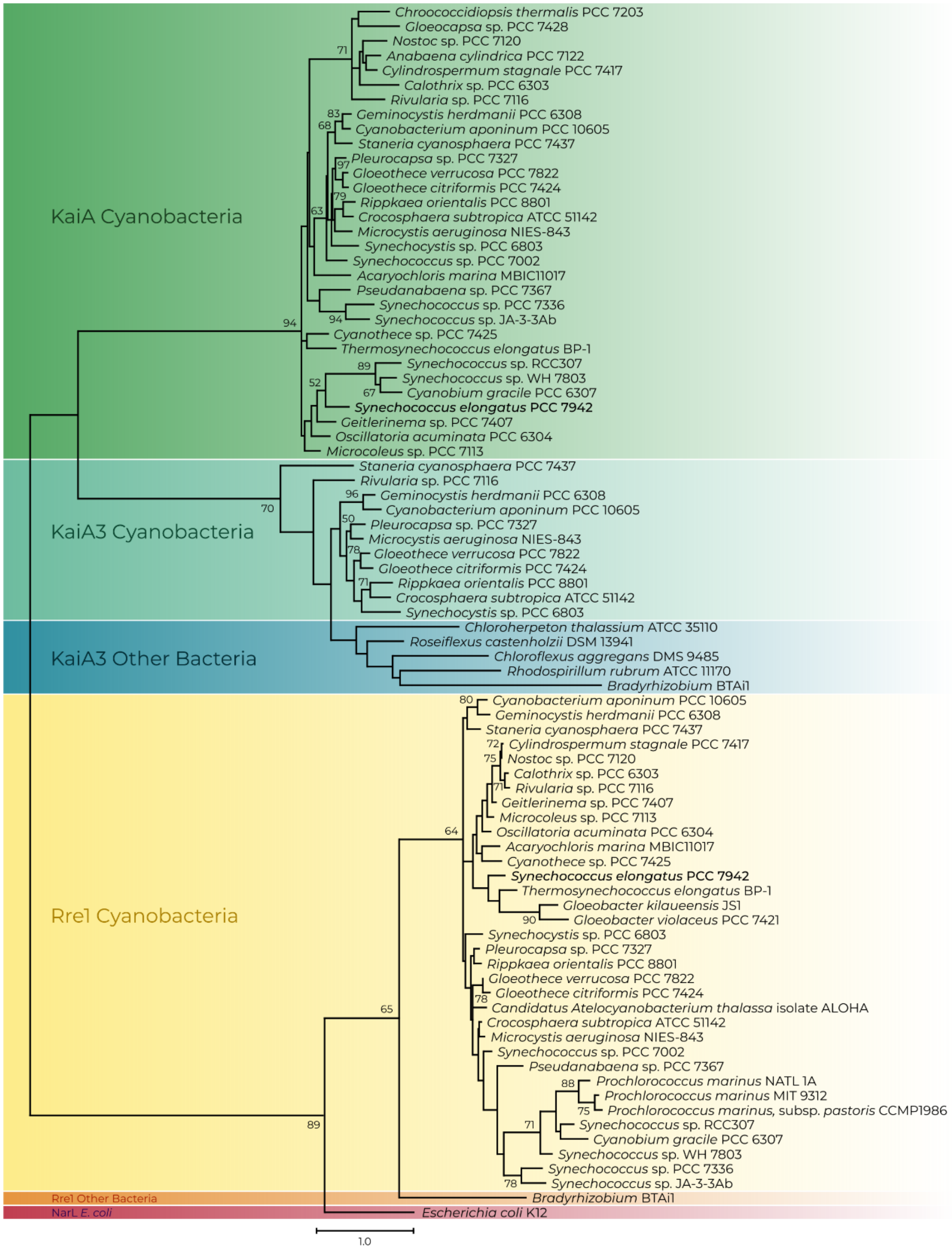

Fig. S3. Maximum likelihood-inferred phylogenetic reconstruction of selected orthologs of Sll0485 (KaiA3), Slr1783 (Rre1), KaiA, and NarL from *E. coli* (UniProtKB–P0AF28). The sequences were aligned with Mafft (L-INS-i default parameters, Jalview), trimmed to position 168 of the C-terminus of the *Synechococcus elongatus* PCC 7942 KaiA. The aligned sequences were used to infer an unrooted maximum-likelihood protein tree. The scale bar indicates one substitution per position. Bootstrap values (n=1000) are displayed at branches. Bootstrap values of less than 50 are not shown.

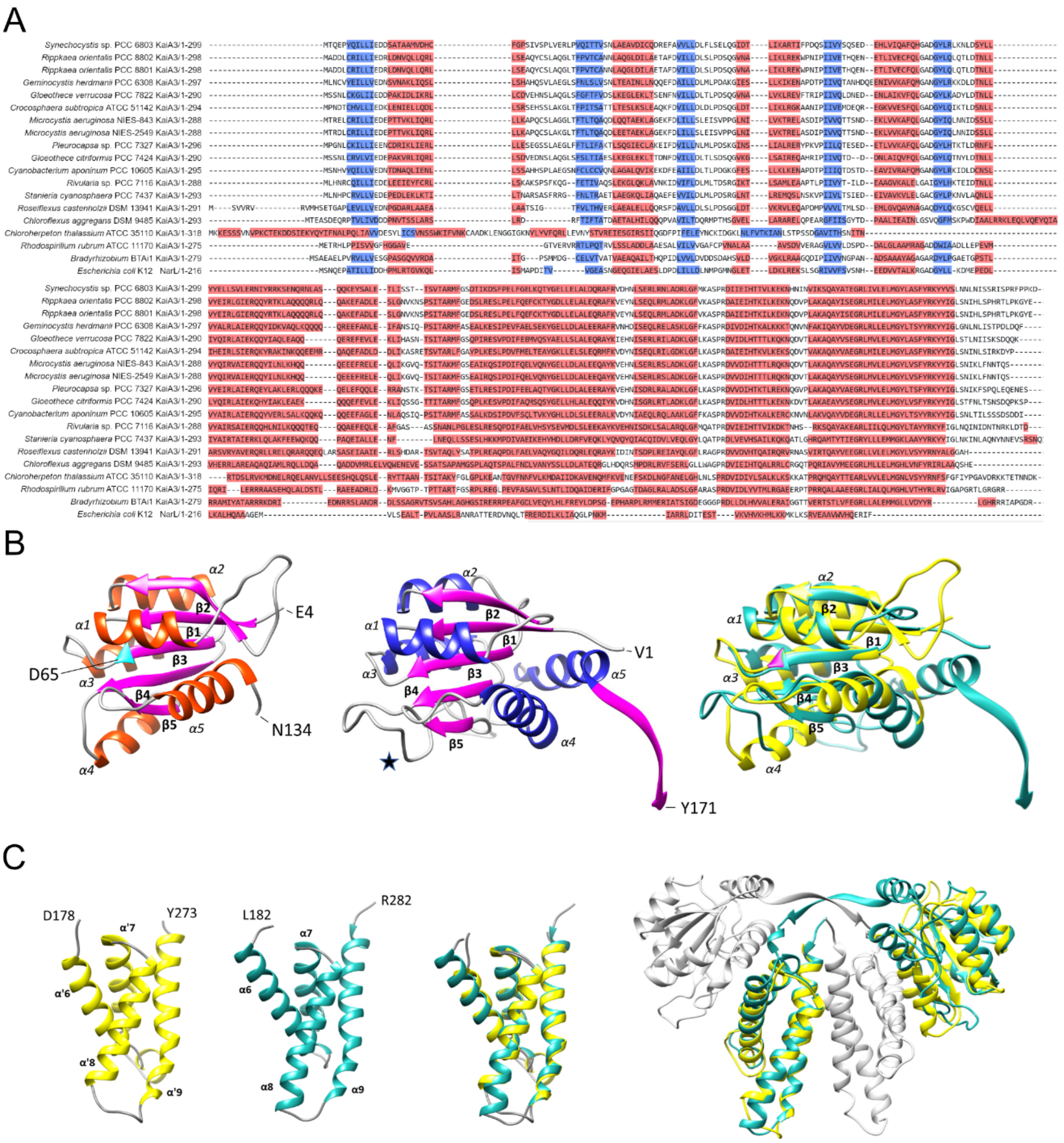

Fig. S4. Secondary structure prediction and modelling of KaiA3. (A) Sequence alignment (Fig. S2) was followed by a secondary structure prediction with Ali2D (preset, 30 % identity cutoff to invoke a new PSIPRED run). Predicted α-helices and β-strands are shown in red and blue, respectively[5]; https://toolkit.tuebingen.mpg.de. (B) The structure of the N-terminal domain of KaiA3 is closely similar to the PsR domain of KaiA. Left: The 3-D structure of the KaiA3 N-terminal domain (template *E. coli* NarL, PDB 1A04) was modelled with SWISS-Model (https://swissmodel.expasy.org/). It comprises residues E4-N134 (initial search with residues 1-140) and displays the canonical fold of a response regulator domain: a five-stranded α/β fold with a central five-stranded parallel β-sheet flanked on both faces by five amphipathic α-helices. The predicted phosphorylation site D65 is shown in turquoise. Middle: The PsR domain of *Synechococcus elongatus* PCC 7942 KaiA (template PDB 4G86, residues 1-171 shown) lacks the α4-helix (highlighted by an asterisk) and a phosphorylation site. Right: The predicted structure of the KaiA3 N-terminus (shown in yellow) superimposes well on the PsR domain of KaiA (shown in light sea green). The putative phosphorylatable aspartate in the KaiA3 β3-sheet is shown in pink. (C) The structure of the C-terminal domain of KaiA3 displays a KaiA-like motif. Left (yellow): The 3-D structure of the KaiA3

C-terminal domain (template KaiA *Thermosynechococcus elongatus* PDB 1V2Z) was modelled with SWISS-Model (https://swissmodel.expasy.org/). It comprises residues D178 – Y273 (initial search with residues 141- 299) and displays a KaiA-like four helix bundle (α'6 - α'9). Left (light sea green): The C-terminal domain of *Synechococcus elongatus* PCC 7942 KaiA (template PDB 4G86, residues 182 - 282). Numbering of the helices according to Ye *et al.*[4]. Middle: Superimposition of the KaiA3 C-terminal domain model structure on the KaiA C-terminus. Right: Superimposition of both KaiA3 domains on the chain B of the KaiA dimer (PDB 4G86). KaiA3 structures are shown in yellow, KaiA structures in light sea green.

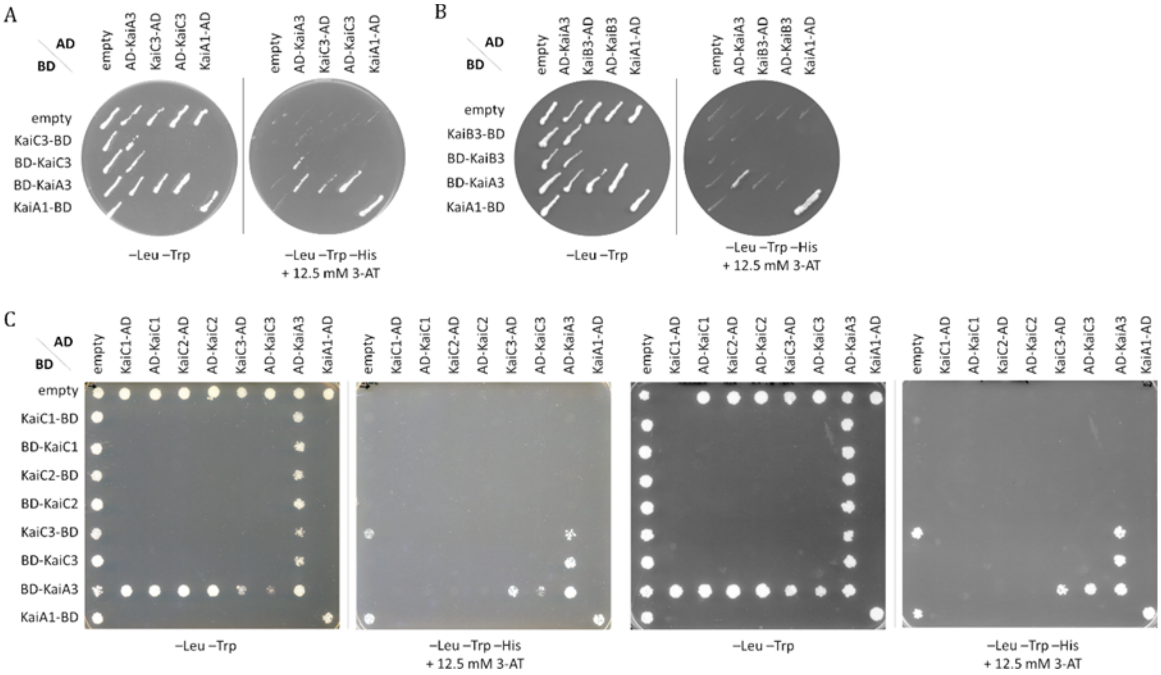

Fig. S5. Complete scans of the plates for KaiA3 interaction analysis with KaiB3 and the three KaiC homologs (KaiC1-KaiC3). Yeast two-hybrid reporter strains carrying the respective bait and prey plasmids, were selected by plating on complete supplement medium (CSM) lacking leucine and tryptophan (-Leu -Trp). As a positive control, *Synechocystis* KaiA1 dimer interaction was used. AD, GAL4 activation domain; BD, GAL4 DNA-binding domain. (A-C) Physical interaction between bait and prey fusion proteins is determined by growth on complete medium lacking leucine, tryptophan and histidine (-Leu -Trp -His) and addition of 12.5 mM 3-amino-1,2,4-triazole 88 (3-AT).

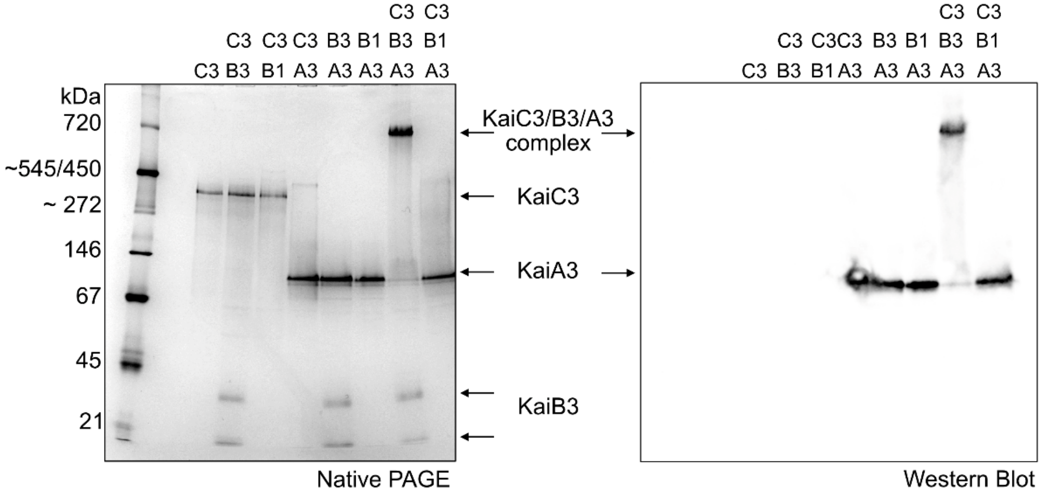

Fig. S6. KaiB1 (B1) does not form a complex with KaiA3 (A3) and KaiC3 (C3). Proteins were incubated for 16 h at 30 °C and subsequently subjected to 4-16% clear native PAGE. The gels were either stained with Coomassie Blue (left, n=3) or blotted and immunodecorated with a monoclonal anti-His antibody for the detection of recombinant KaiA3-His6 (right, n=2). Arrows indicate monomers or protein complexes. The KaiB1 monomer (12 kDa) is not visible in the 4-16% native gradient gel. In contrast to Fig. 2b, a faint band appears in the KaiA3/KaiC3 sample, which is shifted in comparison with the other KaiC3 bands (left). However, KaiA3 is not immunodetected in the shifted band (right).

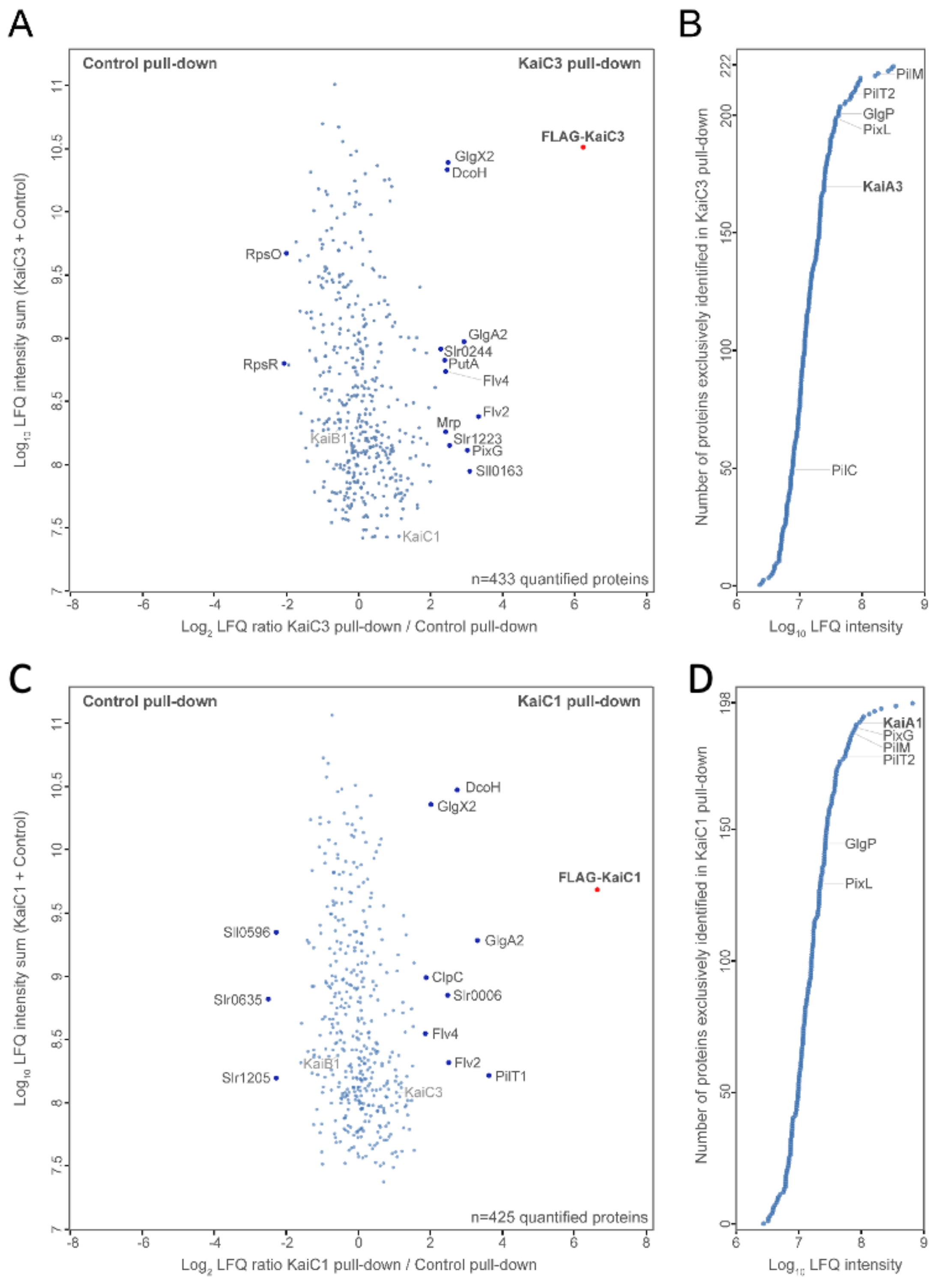

Fig. S7. Immunoprecipitation-coupled LC-MS/MS screening of KaiC3 (A, B) and KaiC1 (C, D) binding partners. Solubilized cell lysates of the WT/FLAG-*kaiC3* (A), WT/FLAG-*kaiC1* (C), and WT/FLAG-*sfGFP* (control) strains cultured under continuous light conditions in copper-depleted BG11 medium were used for α-FLAG co-immunoprecipitation- in pull-down assays. Two independent replicates were measured for each condition. After FLAG-purification, the elution fractions were analyzed by LC-MS/MS. Label-free quantification using the MaxQuant MaxLFQ

algorithm was applied to identify co-enriched proteins. Panels A, C include quantified proteins which were detected in the FLAG-KaiC3 or FLAG-KaiC1 overexpression strain and the control strain. Log_2 LFQ ratios of FLAG-KaiC / control are plotted against the log_{10} LFQ intensity. Significantly enriched proteins (p-value = 0.01, significance B analysis in Perseus software version 1.6.5.0), labeled in dark grey font, are potential interaction partners of KaiC3 or KaiC1. (B, D) Panels include proteins that were exclusively identified in the FLAG-KaiC3 (B) or KaiC1 (D) pull-down assays, but not in the control. Proteins were sorted by their abundance in the KaiC co-immunoprecipitation eluates and selected proteins were labeled. Two independent replicates were measured for each condition. In each replicate, a minimum of two non-redundant peptides were considered for the quantification for each protein group and independent replicates revealed high correlation (Pearson correlation coefficients for LFQ ratios were between 0.74 and 0.89 for KaiC1 pull downs and between 0.67 and 0.9 for KaiC3 pull downs). The mass spectrometry proteomics data were deposited in the ProteomeXchange Consortium (http://proteomecentral.proteomexchange.org) via the PRIDE partner repository, with the dataset identifier PXD042845 and a full list of identified proteins is shown in Supplementary Data S2.

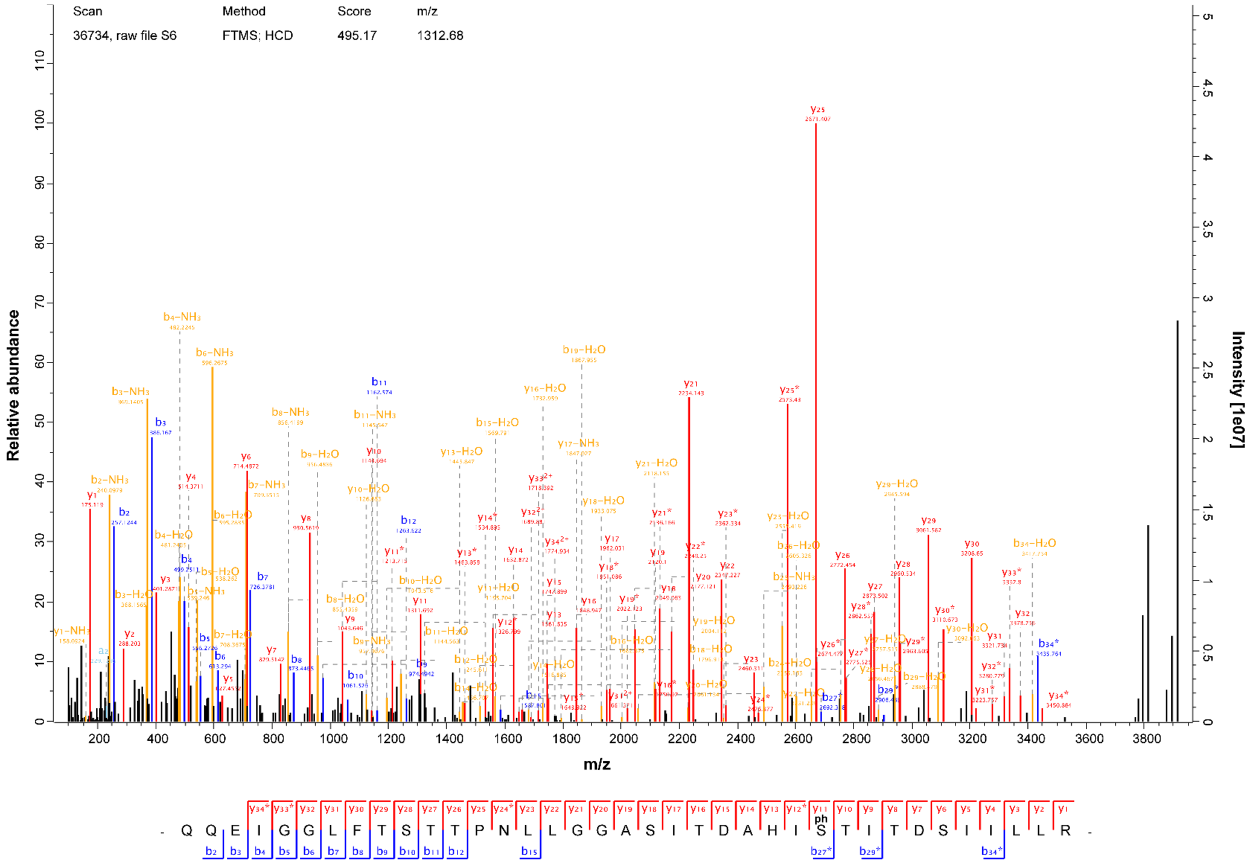

Fig. S8. Representative analysis of phosphopeptides of KaiC3 detected by mass spectrometry. Samples from KaiC3-KaiA3 *in vitro* co-incubation assays (see Materials and Methods KaiC3 phosphorylation in in vitro assays and liquid chromatography mass spectrometry (LC-MS/MS)) were digested with trypsin and analyzed by LC-MS/MS analysis (n=3). Comprehensive b- and y-ion series of an abundant, singly phosphorylated 37 amino acid peptide could be detected, localizing the phosphorylation site on Ser423 (position 27 in the peptide). In multiple cases, phosphorylation could be localized on the neighboring Thr424 position instead. Both sites are homologous positions to the KaiC1 auto-phosphorylation sites Ser432 and Thr433 and appeared with increased abundance after prolonged KaiC3-KaiA3 co-incubation duration.

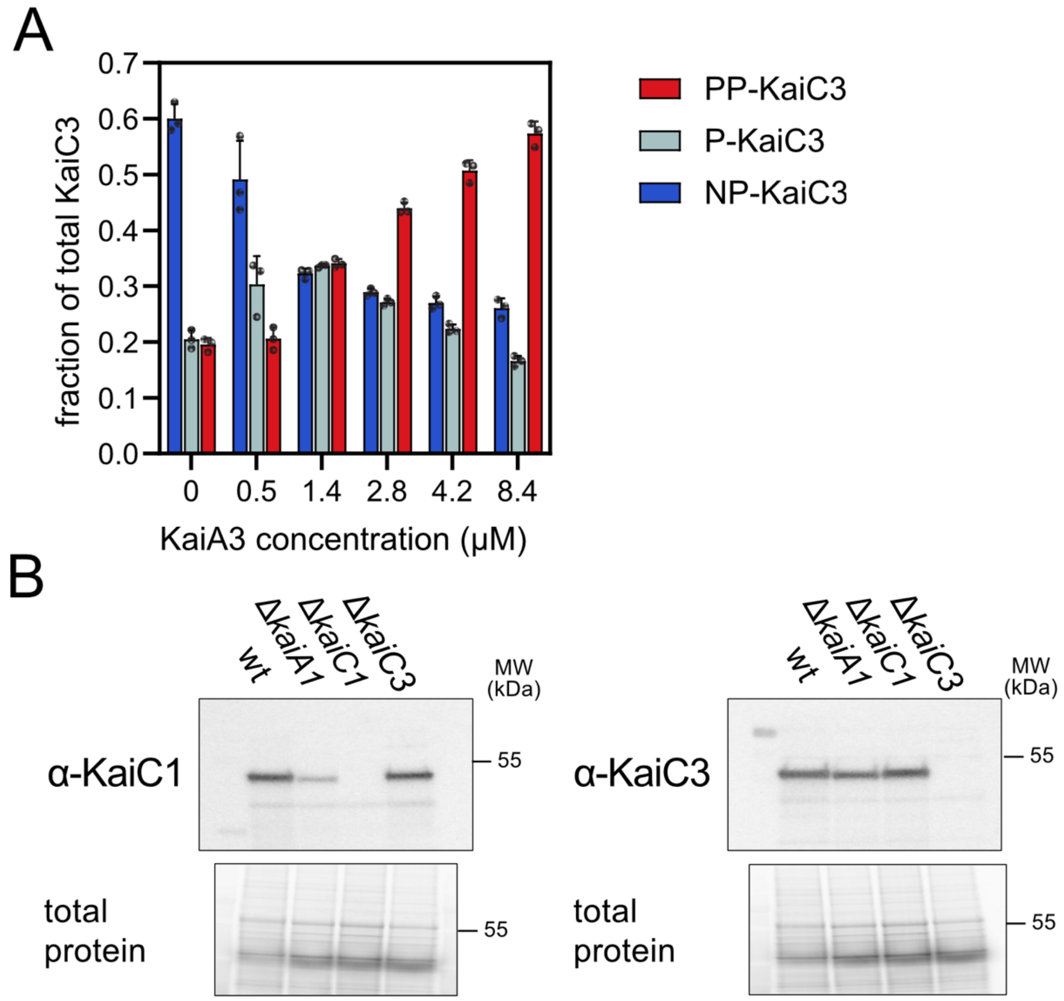

Fig. S9. In vitro and in vivo phosphorylation of KaiC3 (A) Detailed densitometric analysis of KaiC3 phosphorylation after 6 h incubation of 3.4 µM KaiC3 with 7.4 µM KaiB3 and the indicated KaiA3 concentrations. The fractions of double (PP), single (P), and unphosphorylated KaiC (NP-KaiC3) were plotted as average (± SD) (n=3 assays, indicated with dots). Data are derived from the same experiments shown in Fig. 3a,c (in contrast to figure 3c only data from one gel analysis were used for 1.4 µM and 2.8 µM KaiA3). (B) Western blot analysis to check the cross reactivity of KaiC1 and KaiC3 antibodies. Five µg of total protein extracted from wild type (wt), Δ*kaiA1*, Δ*kaiC1*, Δ*kaiC3* cells was loaded per well and western blot was done using KaiC1 specific (αKaiC1) and KaiC3 specific (αKaiC3) antisera. Note that no KaiC1 band was seen in Δ*kaiC1* (left) and no KaiC3 was detected in Δ*kaiC3* cell extracts (right). The bottom panel shows the total protein loaded in stain-free gels (Bio-Rad, USA). This analysis was performed at least two times.

A

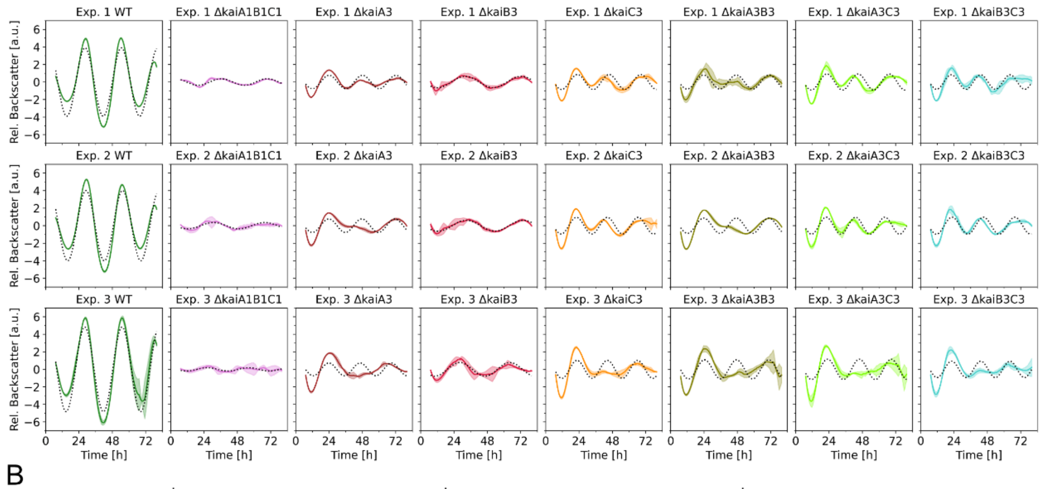

B

	WT			ΔkaiA1B1C1			ΔkaiB3		
	**Exp. 1**	**Exp. 2**	**Exp. 3**	**Exp. 1**	**Exp. 2**	**Exp. 3**	**Exp. 1**	**Exp. 2**	**Exp. 3**
amplitude	3.90	4.00	4.82	0.37	0.36	0.19	0.65	0.62	0.78
angular frequency	0.24	0.24	0.23	0.19	0.17	0.21	0.16	0.15	0.17
phase angle	5.78	5.65	5.99	5.68	1.06	0.75	1.27	1.22	1.37
period	26.21	26.32	26.85	33.14	36.97	29.84	40.20	40.83	36.08
mean_period		*26.46*			*33.32*			*39.04*	
sd_period		*0.34*			*3.57*			*2.58*	

C

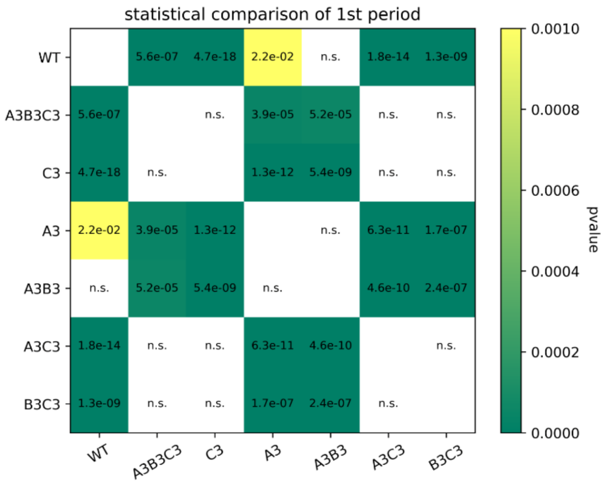

Fig. S10. Detection of circadian output via backscatter measurements. (A) Data from three independent experiments (Exp. 1: All strains except Δ*kaiA1B1C1* n=5 wells, Δ*kaiA1B1C1* n=4 wells, Exp. 2: all strains n=4 wells, Exp 3: all strains n= 5 wells), which were analyzed as described in Fig. 5d-f. Data from Exp. 1 wild type (WT) and Exp. 1 Δ*kaiA1B1C1* are the same as those shown in Fig. 5d and 5e, and are included again for comparison. Solid lines indicate the average of the normalized backscatter with SD (shaded). Curves are smoothed. We attempted to fit the data to a simple harmonic cosine function (dotted line) (B) in A). Only *Synechocystis* WT, Δ*kaiA1B1C1*, and Δ*kaiB3* fit well to the simple harmonic cosine function (dotted line in A). The table lists the parameters of the fit and the derived average period with SD. (C) For the WT and all strains, which were not well described by the simple harmonic cosine function, the period of the first backscattering cycle was calculated (Fig 5j). The heatmap displays the pairwise statistical test. We tested whether the strains had the same variance by using Levene's test. If the variances differed significantly (p-value < 0.05), we performed a Welch T-test. Otherwise, the Student's t-test was performed. Differences with p-value <0.05 were regarded as significant.

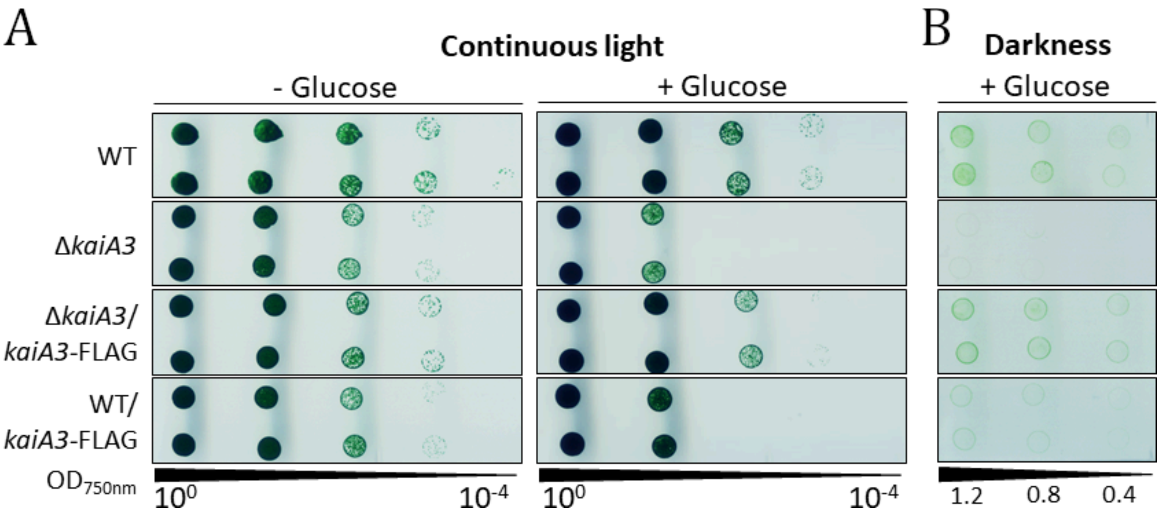

Fig. S11. Overaccumulation of *kaiA3* results in growth defects during mixotrophic and chemoheterotrophic growth. Proliferation of the WT, the *kaiA3* deletion mutant, and the strains Δ*kaiA3*/*kaiA3*-FLAG *and* WT/*kaiA3*-FLAG, expressing *kaiA3* ectopically from a self-replicating plasmid, was tested under phototrophic (continuous light, - glucose), photomixotrophic (continuous light, + glucose) and heterotrophic (darkness, + glucose) conditions. Strains were grown in liquid culture under constant light, different dilutions were spotted on agar plates and incubated under the indicated conditions with 75 µmol photons m^{-2} s^{-1} white light (A) or in darkness (B). A representative result of three independent experiments is shown. (A) Cultures were diluted to OD_{750nm} value 0.4 and dilution series were spotted on agar plates with or without the addition of 0.2% glucose. The plates were analyzed after 6 days of continuous light. (B) Cultures were diluted to OD_{750nm} values of 1.2, 0.8 and 0.4 and spotted on agar plates supplemented with 0.2% glucose. Plates were analyzed after 26 days of darkness. For expression of the *kaiA3*-FLAG gene from the P_{petJ} promoter in the overexpressor strains, all experiments were performed in medium lacking copper.

Table S1.

A. Oligonucleotides used in this study.

Primer	Oligonucleotide Name	Sequence (5‘ – 3‘)	Purpose#
	Construction of yeast two-hybrid expression vectors		
P1	BD-Sll0485-fw	TTGGATCCTACCCAGGAGCCCTACCAAATTC	Y2H
P2	BD-Sll0485-rev	GCACTAGTAGAACTATCTTTGGGGGAAATCG	Y2H
P3	Sll0485-AD-fw	TAGGATCCATGACCCAGGAGCCCTACCA	Y2H
P4	Sll0485-AD-rev	GCCGCTCTAGAAGAACTATCTTTGGGGGAAATC	Y2H
P5	KaiC2-AD-fw	TAGGATCCATGACAGATAACAGCCAAAG	Y2H
P6	KaiC2-AD-rev	GACCTAGGGGGGTTTTGATAAATGTG	Y2H
P7	AD-KaiC2-fw	TAGGATCCATACAGATAACAGCCAAAGTCTC	Y2H
P8	AD-KaiC2-rev	GACTCGAGGGGGTTTTGATAAATGTG	Y2H
P9	BD-KaiC2-fw	TAGGATCCAACAGATAACAGCCAAAGTCTC	Y2H
P10	BD-KaiC2-rev	TACCTAGGGGGGTTTTGATAAATGTG	Y2H
	Construction of *E. coli* expression vectors		
P11	1297_*sll0485*_Nde_fw	AATACATATGACCCAGGAGCCCTA	E
P12	1298_*sll0485*_Xho_rev	TATTCTCGAGAGAACTATCTTTGGGG	E
	Construction of vectors used for deletion and complementation mutants		
P13	pUC19-*sll0485*-fw	GCATTGCCATGGGCAAGAATTCACTGGCCGTC	MU
P14	pUC19-*sll0485*-rev	CCCATTCCTCTGGCGGCAAGCTTGGCGTAATC	MU
P15	US-*sll0485*-fw	GACGGCCAGTGAATTCTTGCCCATGGCAATGC	MU, CP
P16	US-*sll0485*-rev	GACACAACGTGGCTTTCCGTAATCACGGCTAAGTTC	MU
P17	*sll0485*-KmR-fw	CTTAGCCGTGATTACGGAAAGCCACGTTGTGTC	MU
P18	*sll0485*-KmR-rev	AACCTAGGCGATCGGCGAGGTCTGCCTCGTGAAG	MU
P19	DS-*sll0485*-fw	TCACGAGGCAGACCTCGCCGATCGCCTAGGTT	MU, CP
P20	DS-*sll0485*-rev	GATTACGCCAAGCTTGCCGCCAGAGGAATGGG	MU, CP
P21	US-*sll0485*-compl-rev	GTATCAACAGGGACACTTAATCCTCCGGCAAACG	MU
P22	CmR-*sll0485*-compl-fw	TTTGCCGGAGGATTAAGTGTCCCTGTTGATAC	MU
P23	CmR-*sll0485*-compl-rev	GCCTAGGGGATAGCGGCCAGCAATAGACATAAGC	MU
P24	DS-*sll0485*-compl-fw	TTATGTCTATTGCTGGCCGCTATCCCCTAGG	MU
P25	DS-*sll0485*-compl-rev	GATTACGCCAAGCTTGCCTATGAGTTGCCGAGG	MU
P26	pUC19-*sll0485*-compl-rev	CCTCGGCAACTCATAGGCAAGCTTGGCGTAATC	MU
P27	*kaiA3B3*-KmR-rev	GCCTAGGGGATAGCGGGAGGTCTGCCTCGTGAAG	MU
P28	DS-*kaiA3B3*-fw	TCACGAGGCAGACCTCCCGCTATCCCCTAGG	MU
P29	NFLAG-*sll0485*-rev	GGATCCTTAAGAACTATCTTTGGGG	MU, CP
P30	*kaiB3*-AD-rev	GCTCTAGAATCCTCCGGCAAACG	CP
P31	Km-seq-rev	GTATTTCGTCTCGCTCAGGC	CP
P32	Cm-seq-leftout	GCTCCTGAAAATCTCGATAACTC	CP
P33	Km-seg-fw	GCCTGAGCGAGACGAAATAC	CP
P34	NFLAG-*sll0485*-fw	GAATTCACCCAGGAGCCCTAC	MU
P35	pSK9-ORF-fw	CTCCCATAATACCTTCGCGTC	MU, CP

P36	pUR-rev	CTTCCAGATGTATGCTCTTCTGCTC	MU, CP
P37	US-dkaiB3-fw	GACGGCCAGTGAATTCATGACCCAGGAGCCC	MU
P38	US-dkaiB3-rev	GACACAACGTGGCTTTCCCGCTGATAACAAATAAAGCC	MU
P39	dkaiB3-KmR-fw	CTTTATTTGTTATCAGCGGGAAAGCCACGTTGTGTC	MU
P40	pUC19-dkaiB3-fw	AGGGCTCCTGGGTCATGAATTCACTGGCCGTC	MU
P41	*kaiC1* F	AAAATGAACTTACCGATTGTTAACG	MU
P42	*kaiC1* R	AAACTACTCAGCGGTCTTGTCCTT	MU
P43	*kaiC1* ko-Kan F	CTCAAACTACGGGGTACCGTGTCTAAATACATTCAAAT ATGTATCCGCTCATGAG	MU
P44	*kaiC1*-ko Kan R	CTTCATGTGGGTGGTACCGGGATCGAAATCTCGTGATG GC	MU

[#] CP, colony PCR; E, expression; MU, mutagenesis; Y2H, expression in yeast cells.

B. Plasmids used in this study.

Plasmid Name	Description	Reference
pCGADT7ah	Expression of fusion proteins with a C-terminal GAL4$_{(768–881)}$ AD-tag in yeast cells, LEU2, HA epitope tag	Rausenberger *et al.*,[6]
pGADT7ah	Expression of fusion proteins with an N-terminal GAL4$_{(768–881)}$ AD-tag in yeast cells, LEU2, HA epitope tag	Hiltbrunner *et al.,*[7]
pD153	Expression of fusion proteins with a C-terminal GAL4$_{(1–147)}$ DNA-BD-tag in yeast cells, TRP1, c-Myc epitope tag	Shimizu-Sato *et al.*,[8]
pGBKT7	Expression of fusion proteins with an N-terminal GAL4$_{(1–147)}$ DNA-BD-tag in yeast cells, TRP1, c-Myc epitope tag	Clontech, Germany
pGBK-BD-*sll0485*	Expression of Sll0485 with an N-terminal GAL4(1–147) DNA-BD-tag in yeast cells, *TRP1*, c-Myc epitope tag	This study
pGAD-AD-*sll0485*	Expression of Sll0485 with an N-terminal *GAL4*(768–881) AD-tag in yeast cells, *LEU2*, HA epitope tag	This study
pCGAD-*kaiC3*-AD	Expression of KaiC3 with a C-terminal *GAL4*(768–881) AD-tag in yeast cells, *LEU2*, HA epitope tag	Wiegard *et al.,*[9]
pGAD-AD-*kaiC3*	Expression of KaiC3 with an N-terminal GAL4$_{(768–881)}$ AD-tag in yeast cells, LEU2, HA epitope tag	Wiegard *et al.,*[9]
pD153-*kaiC3*-BD	Expression of KaiC3 with a C-terminal GAL4(1–147) DNA-BD-tag in yeast cells, *TRP1*, c-Myc epitope tag	Köbler *et al.*,[10]
pGBKT7-BD-*kaiC3*	Expression of KaiC3 with an N-terminal GAL4(1–147) DNA-BD-tag in yeast cells, *TRP1*, c-Myc epitope tag	Wiegard *et al.,*[9]
pCGAD-*kaiB3*-AD	Expression of KaiB3 with a C-terminal *GAL4*(768–881) AD-tag in yeast cells, *LEU2*, HA epitope tag	Wiegard *et al.,*[9]
pGAD-AD-*kaiB3*	Expression of KaiB3 with an N-terminal *GAL4*(768–881) AD-tag in yeast cells, *LEU2*, HA epitope tag	Wiegard *et al.,*[9]
pCGAD-*kaiA*-AD	Expression of KaiA with a C-terminal *GAL4*(768–881) AD-tag in yeast cells, *LEU2*, HA epitope tag	Köbler *et al.*,[10]
pD153-*kaiA*-BD	Expression of KaiA with a C-terminal GAL4(1–147) DNA-BD-tag in yeast cells, *TRP1*, c-Myc epitope tag	Köbler *et al.*,[10]
pCGAD-*kaiC1*-AD	Expression of KaiC1 with a C-terminal *GAL4*(768–881) AD-tag in yeast cells, *LEU2*, HA epitope tag	Köbler *et al.*,[10]
pGAD-AD-*kaiC1*	Expression of KaiC1 with an N-terminal *GAL4*(768–881) AD-tag in yeast cells, *LEU2*, HA epitope tag	Köbler *et al.*,[10]
pD153-*kaiC1*-BD	Expression of KaiC1 with a C-terminal GAL4$_{(1–147)}$ DNA-BD-tag in yeast cells, *TRP1*, c-Myc epitope tag	Köbler *et al.*,[10]
pGBKT7-BD-*kaiC1*	Expression of KaiC1 with an N-terminal GAL4(1–147) DNA-BD-tag in yeast cells, *TRP1*, c-Myc epitope tag	Wiegard *et al.,*[9]
pCGAD-*kaiC2*-AD	Expression of KaiC2 with a C-terminal *GAL4*(768–881) AD-tag in yeast cells, *LEU2*, HA epitope tag	This study
pGAD-AD-*kaiC2*	Expression of KaiC2 with an N-terminal *GAL4*(768–881) AD-tag in yeast cells, *LEU2*, HA epitope tag	This study
pD153-*kaiC2*-BD	Expression of KaiC2 with a C-terminal GAL4$_{(1–147)}$ DNA-BD-tag in yeast cells, *TRP1*, c-Myc epitope tag	Köbler *et al.*,[10]
pGBKT7-BD-*kaiC2*	Expression of KaiC2 with an N-terminal GAL4(1–147) DNA-BD-tag in yeast cells, *TRP1*, c-Myc epitope tag	This study
pET22-*sll0485*-his6	Expression of Sll0485 with a C-terminal His6 tag in *E. coli* cells	This study
pASK-*kaiC3*	Expression of KaiC3 with an N-terminal Strep-tag (1-11) in *E. coli* cells	Wiegard *et al.,*[9]
pGEX-*kaiB3*	Expression of KaiB3 with an N-terminal GST-tag (1-231) in *E. coli* cells	Wiegard *et al.,*[9]
pGEX-*kaiB1*	Expression of KaiB1 with an N-terminal GST-tag (1-231) in *E. coli* cells	Wiegard *et al.,*[9]
pGEX-*kaiA*7942	Expression of KaiA from *Synechococcus elongatus* PCC 7942 with an N-terminal GST-tag (1-231) in *E. coli* cells	Nishiwaki *et al.*,[11]

pUC19	Cloning vector backbone with multiple cloning site, Amp^R	Norrander *et al.,*[12]
pUC4k	Cloning vector backbone with multiple cloning site, Km^R, Amp^R	Taylor and Rose,[13]
pUC19-Δ*sll0485*	Construction of the Δ*kaiA3* and Δ*kaiA3C3* strains via homologous recombination	This study
pUC19-Δ*sll0485*-compl	Construction of the Δ*kaiA3/kaiA3* complementation strain via homologous recombination	This study
pUC19-Δ*kaiA3B3*	Construction of the Δ*kaiA3B3* and Δ*kaiA3B3C3* strains via homologous recombination	This study
pUR-N-Flag-xyz	pVZ321-based conjugative expression vector, expression of N-terminal FLAG-tagged genes from the copper repressible P*petJ* promotor	Savakis *et al.*,[14]
pUR-NFLAG-*sll0485*	Expression of N-terminal FLAG-tagged *kaiA3*	This study
pUC19_Δ*kaiB3*	Construction of the Δ*kaiB3* and Δ*kaiB3C3* strains via homologous recombination	This study
pJet_Δ*kaiC3*	Construction of the Δ*kaiC3* strain via homologous recombination	Dörrich *et al.,*[15]
pGEMT-Easy	Cloning vector backbone with multiple cloning site, Amp^R	Promega
PsasA-Nina plasmid	Template for amplification of kanamycin resistance	Carl H. Johnson
pGEMT-Δ*kaiC1*	Construction of the Δ*kaiC1* strain via homologous recombination	This study

Supplementary References

1. Waterhouse AM, Procter JB, Martin DMA, Clamp M, Barton GJ. Jalview Version 2-A multiple sequence alignment editor and analysis workbench. *Bioinformatics* **25**, 1189-1191 (2009).

2. Katoh K, Standley DM. MAFFT multiple sequence alignment software version 7: Improvements in performance and usability. *Molecular Biology and Evolution* **30**, 772-780 (2013).

3. Madeira F*, et al.* The EMBL-EBI search and sequence analysis tools APIs in 2019. *Nucleic Acids Research* **47**, W636-W641 (2019).

4. Ye S, Vakonakis I, Ioerger TR, LiWang AC, Sacchettini JC. Crystal structure of circadian clock protein KaiA from *Synechococcus elongatus*. *Journal of Biological Chemistry* **279**, 20511-20518 (2004).

5. Zimmermann L*, et al.* A Completely Reimplemented MPI Bioinformatics Toolkit with a New HHpred Server at its Core. *Journal of Molecular Biology* **430**, 227-2243 (2018).

6. Rausenberger J*, et al.* Photoconversion and Nuclear Trafficking Cycles Determine Phytochrome A's Response Profile to Far-Red Light. *Cell* **146**, 813-825 (2011).

7. Hiltbrunner A*, et al.* Nuclear Accumulation of the Phytochrome A Photoreceptor Requires FHY1. *Current Biology* **15**, 2125-2130 (2005).

8. Shimizu-Sato S, Huq E, Tepperman JM, Quail PH. A light-switchable gene promoter system. *Nature Biotechnology* **20**, 1041-1044 (2002).

9. Wiegard A*, et al. Synechocystis* KaiC3 displays temperature- And KaiB-dependent ATPase activity and is important for growth in darkness. *J Bacteriol* **202**, 1-36 (2020).

10. Köbler C, Schultz SJ, Kopp D, Voigt K, Wilde A. The role of the *Synechocystis* sp. PCC 6803 homolog of the circadian clock output regulator RpaA in day–night transitions. *Molecular Microbiology* **110**, 847-861 (2018).

11. Nishiwaki T*, et al.* Role of KaiC phosphorylation in the circadian clock system of Synechococcus elongatus PCC 7942. *Proc Natl Acad Sci U S A* **101**, 13927-13932 (2004).

12. Norrander J, Kempe T, Messing J. Construction of improved M13 vectors using oligodeoxynucleotide-directed mutagenesis. *Gene* **26**, 101-106 (1983).

13. Taylor LA, Rose RE. A correction in the nucleotide sequence of the Tn9O3 kanamycin resistance determinant in pUC4K. *Nucleic Acids Research* **16**, 358 (1988).

14. Savakis P*, et al.* Light-induced alteration of c-di-GMP level controls motility of *Synechocystis* sp. PCC 6803. *Molecular Microbiology* **85**, 239-251 (2012).

15. Dörrich AK, Mitschke J, Siadat O, Wilde A. Deletion of the *Synechocystis* sp. PCC 6803 *kaiAB1C1* gene cluster causes impaired cell growth under light-dark conditions. *Microbiology* **160**, 2538-2550 (2014).
