# Supplementary material for: Two KaiABC systems control circadian oscillations in one cyanobacterium
Source: Nat Commun. 2024 Sep 3;15:7674. doi: 10.1038/s41467-024-51914-5 (PMC11372060; doi:10.1038/s41467-024-51914-5)
Supplement: Supplementary file 3 — Description of Additional Supplementary Files [file 41467_2024_51914_MOESM3_ESM.pdf]

Description of Additional Supplementary Files:

Supplementary Dataset 1: (separate Excel file). Putative orthologs of KaiA3 in cyanobacteria and prokaryotes. Header names are described in the following and the exact name is mentioned in parenthesis. Information is provided regarding the organism (name), corresponding genus (genus), taxonomy (taxonomy), and taxonomic identifier (taxid). Furthermore, the annotated protein name on NCBI (protein), the protein identifier on NCBI (protein_id), the genome identifier where the protein originated from (genome_id), the date when it was last modified on NCBI (date), BLAST statistics (e_value, bitscore, identity), the length of the protein (length) as well as the sequence (seq) were recorded. In addition, the protein id of backward best hit from Synechocystis (synechocystis_prot_id) as well as the genome identifier for the genome assembly (synechocystis_id) was stored.

Supplementary Dataset 2: (separate Excel file). Dataset from immunoprecipitation-coupled LC-MS/MS analyses of KaiC3 and KaiC1 interactome analyses. Identified and quantified proteins from label-free analysis of α-FLAG-KaiC3 or - KaiC1 and control co-immunoprecipitation are listed.

Supplementary Dataset 3: (separate Excel file). Dataset of KaiA3B3C3 in vitro co-incubation assays on KaiC3 phosphorylation. Localized KaiC3 phosphorylation sites and phosphorylation occupancies of Ser423/Thr424 are listed.

Supplementary Dataset 4: (separate zip folder). Raw data and protocols of backscatter experiments shown in Fig. 5.
