# Supplementary material for: Two KaiABC systems control circadian oscillations in one cyanobacterium
Source: Nat Commun. 2024 Sep 3;15:7674. doi: 10.1038/s41467-024-51914-5 (PMC11372060; doi:10.1038/s41467-024-51914-5)
Supplement: Supplementary file 8 — Reporting Summary [file 41467_2024_51914_MOESM8_ESM.pdf]

nature portfolio

Corresponding author(s): Annegret Wilde, Ilka Axmann

Last updated by author(s): Jul 29, 2024

Reporting Summary

Nature Portfolio wishes to improve the reproducibility of the work that we publish. This form provides structure for consistency and transparency in reporting. For further information on Nature Portfolio policies, see our Editorial Policies and the Editorial Policy Checklist.

Statistics

For all statistical analyses, confirm that the following items are present in the figure legend, table legend, main text, or Methods section.

n/a	Confirmed	
☐	☒	The exact sample size (*n*) for each experimental group/condition, given as a discrete number and unit of measurement
☐	☒	A statement on whether measurements were taken from distinct samples or whether the same sample was measured repeatedly
☐	☒	The statistical test(s) used AND whether they are one- or two-sided<br>*Only common tests should be described solely by name; describe more complex techniques in the Methods section.*
☒	☐	A description of all covariates tested
☒	☐	A description of any assumptions or corrections, such as tests of normality and adjustment for multiple comparisons
☐	☒	A full description of the statistical parameters including central tendency (e.g. means) or other basic estimates (e.g. regression coefficient) AND variation (e.g. standard deviation) or associated estimates of uncertainty (e.g. confidence intervals)
☐	☒	For null hypothesis testing, the test statistic (e.g. *F*, *t*, *r*) with confidence intervals, effect sizes, degrees of freedom and *P* value noted<br>*Give P values as exact values whenever suitable.*
☒	☐	For Bayesian analysis, information on the choice of priors and Markov chain Monte Carlo settings
☒	☐	For hierarchical and complex designs, identification of the appropriate level for tests and full reporting of outcomes
☒	☐	Estimates of effect sizes (e.g. Cohen's *d*, Pearson's *r*), indicating how they were calculated

Our web collection on statistics for biologists contains articles on many of the points above.

Software and code

Policy information about availability of computer code

Data collection	Backscatter raw data were acquired in a a BioLector XT microbioreactor equipped with a Light Array Module (LAM) (Beckmann Coulter).<br>nanoLC-MS/MS analysis was performed on a Q Exactive HF mass spectrometer (Thermo Fisher Scientific) operated in data-dependent acquisition mode.<br>Protein bands after Coomassie staining or immunodetection were visualized using a Fusion SL chemiluminescence detector (Vilber Lourmat), ChemiDoc MP Imaging System (Bio-Rad) or ChemiDoc XRS+ Imaging System.<br>Backscatter raw data were acquired in a a BioLector XT microbioreactor equipped with a Light Array Module (LAM) (Beckmann Coulter).<br>nanoLC-MS/MS analysis was performed on a Q Exactive HF mass spectrometer (Thermo Fisher Scientific) operated in data-dependent acquisition mode.<br>Protein bands after Coomassie staining or immunodetection were visualized using a Fusion SL chemiluminescence detector (Vilber Lourmat), ChemiDoc MP Imaging System (Bio-Rad) or ChemiDoc XRS+ Imaging System.
Data analysis	Reciprocal BLAST and co-occurance analysis was performed as described by Schmelling et al.27 using Python. The script is available at https://github.com/schmelling/reciprocal_BLAST/blob/master/notebooks. The conservation of gene order was analysed using the web tool 'SyntTax'89; https://pubmed.ncbi.nlm.nih.gov/23323735/.<br>Multiple sequence alignments were performed with Mafft and Jalview. 'Ali2D' was used for secondary structure prediction92 https://toolkit.tuebingen.mpg.de). Three-dimensional protein structures were modeled using either Phyre2 or SWISS-MODEL93, 94 (http://www.sbg.bio.ic.ac.uk/phyre2/html/page.cgi?id=index; https://swissmodel.expasy.org/). The resulting structures were analysed and illustrated using UCSF Chimera95 (https://www.cgl.ucsf.edu/chimera/).<br>Phylogenetic reconstruction of the protein trees was achieved with MEGA X96, 97.

Backscatter data were analysed using Python (version 3.9.16), with packages: pandas (version 1.5.3), matplotlib (version 3.7.1), regex (version 2022.7.9), numpy (version 1.24.3), scipy (version 1.10.1), scikit-learn (version 1.2.2). The script was modified from Berwanger et al. 2023 and is available at https://github.com/flo-sti/cyano-backscatter.
Raw data after LC-MS/MS were processed using the MaxQuant software (version 1.5.2.8)
GraphPad Prism 9.5.1 was used for statistical analysis of relative KaiC phosphorylation derived from gel or western blot analysis.

For manuscripts utilizing custom algorithms or software that are central to the research but not yet described in published literature, software must be made available to editors and reviewers. We strongly encourage code deposition in a community repository (e.g. GitHub). See the Nature Portfolio guidelines for submitting code & software for further information.

Data

Policy information about availability of data

All manuscripts must include a data availability statement. This statement should provide the following information, where applicable:

- Accession codes, unique identifiers, or web links for publicly available datasets
- A description of any restrictions on data availability
- For clinical datasets or third party data, please ensure that the statement adheres to our policy

Raw and processed data generated in this study are available on figshare:
Raw data: https://doi.org/10.6084/m9.figshare.25218143
Processed Data: https://doi.org/10.6084/m9.figshare.25218137, Alignments: https://doi.org/10.6084/m9.figshare.25218122, Phylogeny: https://doi.org/10.6084/m9.figshare.25218134), processed KaiA3 hits are also available as Supplementary Data S1.
The mass spectrometry proteomics data generated in this study have been deposited in the ProteomeXchange Consortium database (http://proteomecentral.proteomexchange.org) via the PRIDE partner repository 120, with the dataset identifier PXD042846 (analysis of KaiC3 phosphorylation) https://ftp.pride.ebi.ac.uk/pride/data/archive/2024/07/PXD042846, PXD042845 (screening of KaiC3 and KaiC1 binding partners) https://ftp.pride.ebi.ac.uk/pride/data/archive/2024/07/PXD042845, and summarized data are available as Supplementary Data S2 and S3.
Datasets S1 to S4 are available as Supplementary Data.

Source data are provided with this paper.

A source data file containig original image files and raw data was submitted together with the manuscript.

Research involving human participants, their data, or biological material

Policy information about studies with human participants or human data. See also policy information about sex, gender (identity/presentation), and sexual orientation and race, ethnicity and racism.

Reporting on sex and gender	not applicable
Reporting on race, ethnicity, or other socially relevant groupings	not applicable
Population characteristics	not applicable
Recruitment	not applicable
Ethics oversight	not applicable

Note that full information on the approval of the study protocol must also be provided in the manuscript.

Field-specific reporting

Please select the one below that is the best fit for your research. If you are not sure, read the appropriate sections before making your selection.

☒ Life sciences ☐ Behavioural & social sciences ☐ Ecological, evolutionary & environmental sciences

For a reference copy of the document with all sections, see nature.com/documents/nr-reporting-summary-flat.pdf

Life sciences study design

All studies must disclose on these points even when the disclosure is negative.

Sample size	This work does not involve the analysis of populations. For functional assays, experimentes were repeated as stated in the mansucript.
Data exclusions	No data was excluded

Replication	The number of independent biological replicates is stated in each figure legend. Technical and biological variability was not conflated. For data points with less than 3 replicates no statistics was derived.
Randomization	Randomization was not relevant because our study did not involve the allocation of samples/organisms/participants into experimental groups.
Blinding	Group allocation was not involved in our study. Investigators were not blinded during data collection because the nature of collected data. Interpretation of data was performed based on quantification of results (e.g. bands in gels). Subjective interpretations were avoided by quantification.

Reporting for specific materials, systems and methods

We require information from authors about some types of materials, experimental systems and methods used in many studies. Here, indicate whether each material, system or method listed is relevant to your study. If you are not sure if a list item applies to your research, read the appropriate section before selecting a response.

Materials & experimental systems

n/a	Involved in the study	
☐	☒	Antibodies
☒	☐	Eukaryotic cell lines
☒	☐	Palaeontology and archaeology
☒	☐	Animals and other organisms
☒	☐	Clinical data
☒	☐	Dual use research of concern
☒	☐	Plants

Methods

n/a	Involved in the study	
☒	☐	ChIP-seq
☒	☐	Flow cytometry
☒	☐	MRI-based neuroimaging

Antibodies

Antibodies used	• monoclonal anti-6x-His Tag antibody conjugated to HRP (MA1-21315-HRP, Thermo Fisher, LOT number YH374751)<br>• anti-KaiC1 antibody, raised against synthetic peptide NLPIVNERNRPDVPRKGVQ coupled to keyhole limpet haemocyanin. Affinity-purified monospecific IgG fraction. Produced by Pineda Antikörper services. Published in Wiegard et al. 2013, https://doi.org/10.1099/mic.0.065425-0<br>• anti-KaiC3 antibody, raised against synthetic peptide MYTAQSEVERLSGLFDEKI coupled to keyhole limpet haemocyanin. Whole IgG fraction. Produced by Pineda Antikörper services. Published in Wiegard et al. 2013, https://doi.org/10.1099/mic.0.065425-0<br>• Goat anti-rabbit IgG (H+L) Secondary Antibody, HRP (31460, Thermo Fisher)<br>• anti-rabbit αHRP antibody (Thermo Fisher Scientific Inc., USA)
Validation	anti-KaiC1 and anti-KaiC3 antibodies were validated by western blot analysis using respective mutant strains and expressed proteins (see Figure S9b). For commercial His antibody we refer to the respective websites of the supplier (Thermo Fisher, https://www.thermofisher.com/antibody/product/6x-His-Tag-Antibody-clone-HIS-H8-Monoclonal/MA1-21315-HRP).

Plants

Seed stocks	not applicable
Novel plant genotypes	not applicable
Authentication	not applicable
